# Dynamics of Specific Anti-*Mycobacterium avium* Subsp. *paratuberculosis* Antibody Response through Age

**DOI:** 10.1371/journal.pone.0063009

**Published:** 2013-04-29

**Authors:** Søren Saxmose Nielsen, Nils Toft, Hisako Okura

**Affiliations:** Department of Large Animal Sciences, University of Copenhagen, Frederiksberg C, Denmark; Universita di Sassari, Italy

## Abstract

*Mycobacterium avium* subsp. *paratuberculosis* (MAP) causes a chronic infection in cattle. MAP infected cattle with humoral immune (HI) reactions with IgG antibodies are usually those where latency of infection has ceased and their infection is progressing towards reduced milk yield, weight loss and significant bacterial excretion in feces. The proportion of detectable infections among all infected animals that will develop disease is often referred to as ‘the tip of the iceberg’. The purpose of this study was to estimate this proportion. Test-records from 18,972 Danish dairy cows with MAP specific IgG antibodies on their final test-record were used to estimate age-specific sensitivities (Se). These cows were the infected ones considered to develop disease in a population with a representative age-distribution and were defined as cases. The specificity (Sp) of the test was estimated based on test-results from 166,905 cows, which had no MAP IgG antibodies in their final four test-records. The Sp, age-specific Se and maximum Se were used to estimate the probability of having HI at a given age resulting in the proportion of infected cows with HI at a given age. For cows 2 years of age, the proportion of detectable cases was 0.33, while it was 0.94 for cows 5 years of age. Thus, there was a significant shift in the tip of the iceberg with aging. This study provided a model for estimating the proportion of latent chronic infections that would progress to disease, and the results can be used to model infection dynamics.

## Introduction

Paratuberculosis is a chronic infection of cattle and other ruminants, caused by *Mycobacterium avium* subsp. *paratuberculosis* (MAP) [1]. MAP infections are presumed to occur in calfhood with a subsequent latent infection, where pro-inflammatory immune responses are thought to keep the infection under control [2], [3]. In few animals, the infection evolves before 2 years of age, but from 2 to 6 years of age, a large proportion of infected animals appears to develop anti-inflammatory immune responses characterized by IgG antibodies [4], [5]. Latency is a common feature of mycobacterial infections and disease activation may be due to immunosuppression [6]. Occurrence of IgG antibodies usually precedes the primary adverse effects of the MAP infection, namely reduced milk yield, reduced body weight and major bacterial excretion [7], [8], [9]. The animals that will experience these losses are also those of primary interest in financial models assessing the impact of the infection. Not all infected animals will experience progression of the infection, either because they will be culled early or because they are resistant to disease. Resistance is so far insufficiently characterized but seems to occur [10]. Therefore, a population may consist of three groups of animals: 1) non-infected or potentially latently infected animals where infection will never evolve; 2) MAP infected animals with a latent infection that will evolve within the expected life-time; and 3) MAP infected animals where the infection is progressing with a predominant anti-inflammatory or humoral immune response (HI). The size of these three groups is of interest when disease progression should be predicted, for example in mathematical infection models [11], [12], and the proportion of those with HI among all infected, where the infection will develop, is often referred to as “the tip of the iceberg”. It has previously been suggested, that 50 to 70% of MAP infected animals comprise the invisible part of the iceberg [13], but no evidence supporting this claim was provided. Furthermore, this proportion would most likely depend on the age-distribution in the population, because the infection is chronic.

Based on the assumptions that: 1) animal are infected in calfhood or no later than the start of 1^st^ lactation (usually after the age of 2 years); and 2) if IgGs are present, then the cow has HI; then the proportion of cattle with HI can be estimated. The purpose of this study was to, at different ages, estimate the proportion of MAP infected cattle with HI among all MAP infected animals where infection progresses from a stage of latency to a stage with HI.

## Materials and Methods

### Study Design, Herds and Animals

The study was performed as a retrospective, longitudinal study. All data were retrieved from the Danish Cattle Database (Knowledge Centre for Agriculture, Aarhus, Denmark). The data were collected from all 834 dairy herds participating in the voluntary Danish control program on bovine paratuberculosis [14] throughout the period 15 October 2008 to 27 September 2012. Milk samples were collected from all lactating cows four times per herd per year. Minimum herd contribution was 116 samples, median was 1,528 and maximum was 12,801 samples. A total of 1,913,916 samples were initially included in the study. Due to the observational study type, the number of samples per animal differed with a lower quartile of 3 samples, median of 4 samples and upper quartile of 6 samples per animal. Sixty-eight per cent of the samples were from Danish Holsteins, 17 per cent were from Danish Jerseys, and the remaining samples were from either mixed or minor dairy breeds. The parity distribution at sampling was as follows: parity 1: 47%; parity 2: 27%; parity 3: 14%; parity 4: 7%; parity 5: 3%; and parity >5: 2%.

### Diagnostic Testing

The milk samples were collected as part of the routine milk recording scheme and sent to Eurofins Stein’s Laboratory (Holstebro, Denmark). Samples were treated with the preservative bronopol and shipped to the laboratory within 6 hours after the final sample was obtained. Samples were then tested using the commercial ID Screen® Paratuberculosis Indirect ELISA screening kit according to manufacturer instructions (ID Vet, Montpellier, France). The test is a *M. phlei* absorbed ELISA detecting IgG. Sample results were recorded as a sample-to-positive ratio (S/P), and were subsequently dichotomized using an S/P of 0.15 as recommended by the manufacturer. Samples collected from cows 0 to 5 days after calving were excluded, because of potential false-positive reactions in these samples [15], and because farmers are recommended not to use these samples for MAP testing.

### Target Condition and Case Definition

The target condition was MAP infected animals, where infection would progress to HI within the expected life-time of an animal. Data were divided into cases for estimation of sensitivity and non-cases for estimation of specificity. The target condition describes the underlying condition that we wish to make inference about, whereas a case definition is the practical realization of this condition.

**Figure 1:**
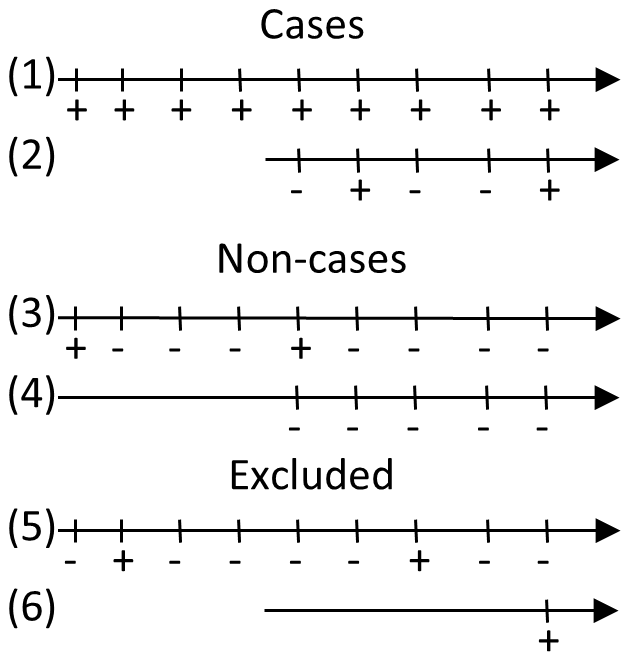
Examples of cases, non-cases and excluded cows. The grey rectangle shows the part of the tests used for case and non-case definition, while the rest of the test results were used for the estimations. Nos. 1 and 2 are cases because last sample is positive. Nos. 3 and 4 are non-cases, because last four samples were negative. No. 5 is excluded because it does not fulfill criteria of last sample being positive (cases) or last 4 samples being negative (non-cases). No. 6 would be excluded, because there was only one sample, and case positivity and test positivity would be identical.

A case was defined as a cow, which was test-positive in the ID Screen test at the last sample of minimum two samples. This case definition should firstly reflect the target condition and ultimately proof of progression from “infection to disease”. This definition was challenged in a sensitivity analysis (see later). Cows with only one sample were excluded, because the case occurred at the time of testing (Figure 1). A total of 18,972 cows fulfilled these criteria and contributed 94,597 test-results. Because we used all animals in the program, and because we used the last sample of each cow, we assumed that the expected life-time of the infected animals would correspond more or less to the resulting dataset.

A non-case was defined as a cow having at least 5 samples with the last 4 samples being negative. A total of 166,905 cows fulfilled this criterion. This definition was also challenged in a sensitivity analysis (see later).

### Descriptive Statistics

Descriptive statistics was done by cross-tabulation of ELISA-responses stratified by age for cases and non-cases. Furthermore, the proportion of test-positive samples after the first positive sample was estimated for each case cow, and the cows were divided into five groups based on this proportion: 1) <25% of samples were positive; 2) 25–49% of samples were positive; 3) 50–74% of samples were positive; 4) 75–99% samples were positive; and 5) all samples were positive, after the first positive sample. The S/P-value at different time-points relative to the first positive test was then estimated using a generalized additive model [16]: S/P = β_0_+ F(Time) for each of the above-mentioned groups, where S/P was the recorded S/P value in the ELISA, β_0_ was the base-line ELISA-reaction and F(Time) was the smoothing function of the effect of time in years relative to the first date of testing positive. The purpose of these profiles was purely descriptive.

### Estimation of Sensitivity, Specificity and Proportion of Cows with HI and CMI

Age-specific sensitivities were subsequently estimated among case cows using non-linear logistic regression with age as a covariate. The model was:
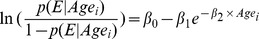
where p(E|Age*_i_*) was the probability of testing positive in the ELISA ( = sensitivity) at Age*_i_*, Age*_i_* was the age in years at testing, and β_0_ was the upper limit of the logit of Se at maximum age, β_1_ the scale factor and β_2_ the coefficient for the decay of the effect of age. Only samples from cows less than 10 years of age were included in further analyses, and a random observation was selected for each cow to avoid clustering in sensitivity estimation.

Specificity was estimated as the proportion of test-negatives among non-cases, with the uncertainty computed as an asymptotic 99% confidence interval (CI) for binomial data.

A sensitivity analysis was carried out to determine if the definition of non-cases affected the estimation of specificity: the number of samples required to be defined as a non-case was changed to 5 and 6 samples. Likewise, the effect of changing the definition used for cases was investigated by changing the definition of case to be a cow with the last two samples as test-positive instead of only one.

The probability of testing positive in the antibody ELISA for cows without infection or with life-long latent infection was defined as 1– specificity, because these cows do not have IgG antibodies. The proportion *q* of cows with HI at a specific age was then estimated as:
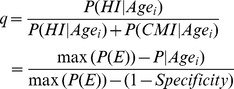
where max(p(E)) was the maximum sensitivity, or the proportion of cows that eventually developed IgG antibodies.

## Results

**Figure 2:**
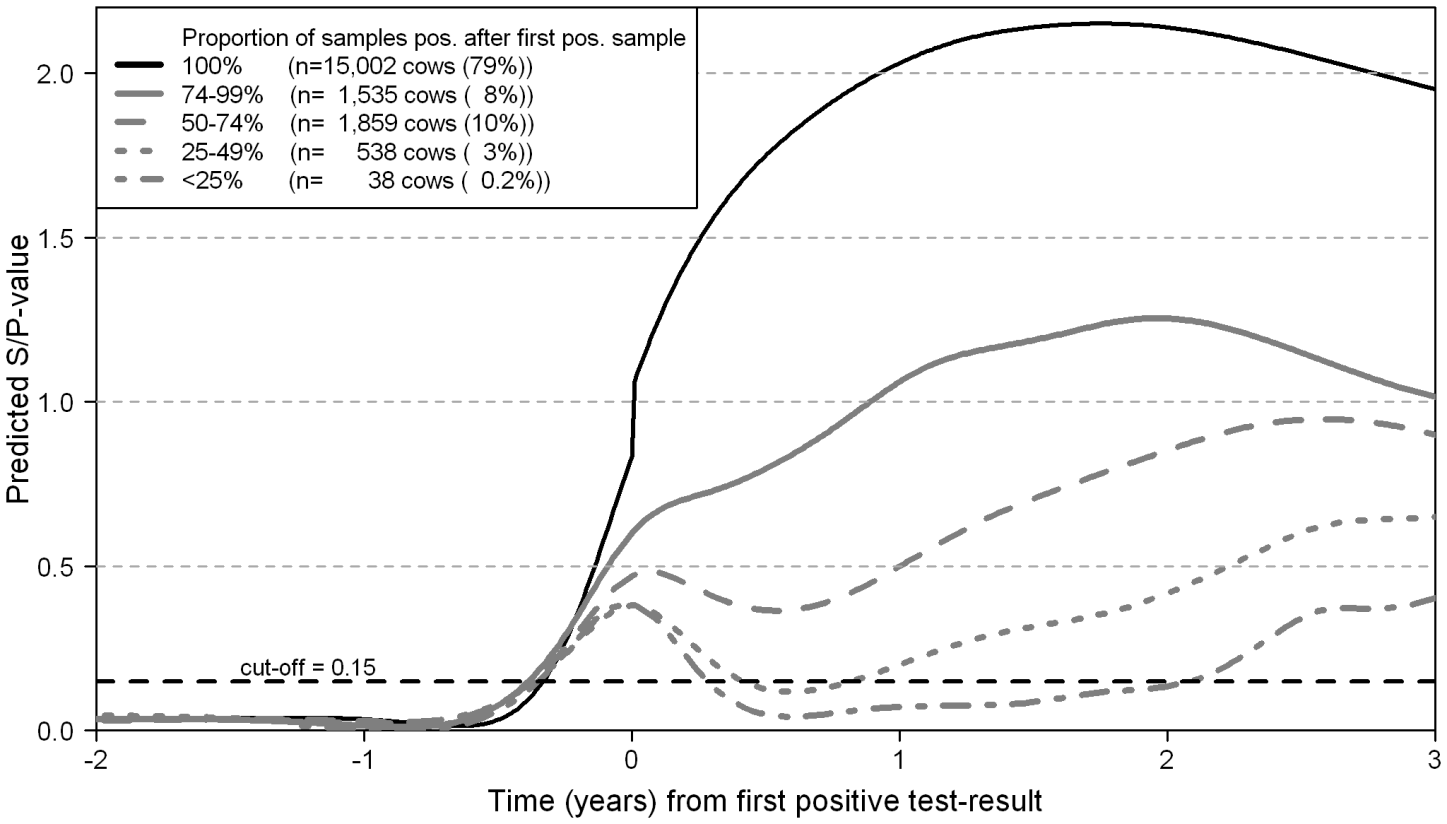
Smoothed test-responses for the case cows used for sensitivity estimation. Test-responses are relative to the first date the animal tested positive. The black line represents the 79% of cows where all subsequent test-results were positive, whereas the grey lines represent the remaining 21% where different proportions of the total number of samples were test-positive.

Descriptive statistics with age-distributions at testing and ELISA test-results for cases and non-cases are shown in Table 1. The median age at the last test for cases was 4.73 years, with the interquartile range being 3.86 to 5.76 years. The predicted S/P-values relative to first day the cow tested positive are shown in Figure 2, stratified by the proportion of test-positive samples after the first positive sample. The distribution of cows with different proportions of test-positive samples is also shown, e.g. 79% of cows had all samples positive after the first positive sample, with only 0.2% having less than 25% of samples positive.

**Table 1:** Distribution of ELISA positives among cases and non-cases in different age-groups.

		Age-group^1^ (%)						
Cases		<2	2–3	3–4	4–5	5–6	6–7	>7
ELISA	+	23(28)	2323(43)	3361(62)	2772(71)	1755(76)	833(76)	594(77)
	−	60(72)	3094(57)	2047(38)	1106(29)	559(24)	270(24)	175(23)
**Non-cases**								
ELISA	+	0	23(1.1)	780(1.2)	617(1.2)	386(1.5)	211(1.7)	212(2.0)
	−	0	2125(98.9)	65458(98.8)	49514(98.8)	24722(98.5)	12438(98.3)	10419(98.0)

1 Age in years.

One randomly selected observation from each cow was used.

The specificity was estimated to 0.9866 (99% CI: 0.9859–0.9874). Changing the number of samples required for being defined as a non-case from 4 to 5 or 4 to 6 changed the specificity by 0.0001 and 0.0002, respectively.

**Figure 3:**
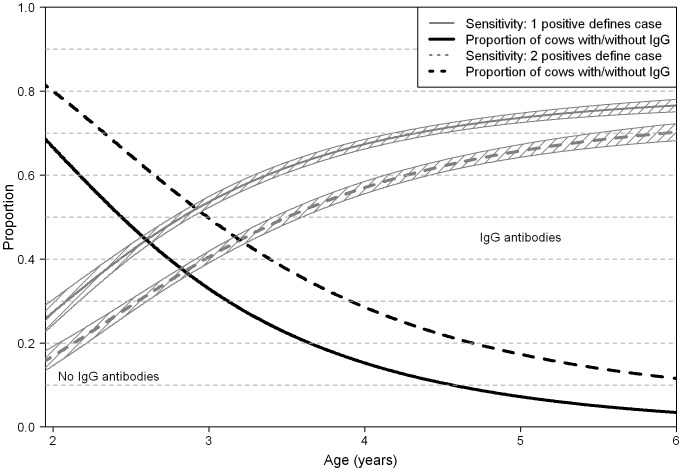
Age-specific sensitivities and proportion of cows with pro- and anti-inflammatory responses. Shaded area illustrates 95% point specific confidence interval of sensitivities. The proportion of cows with and without IgG antibodies at different ages above and below the dotted line, respectively. The full lines represent the data based on cases defined as the last sample being positive, whereas the dotted lines represent data where cases were required to be positive on the last two samples.

The parameters in the regression model was estimated to: β_0_: 1.32 (standard error (SE): 0.057), β_1_: −0.70 (SE: 0.051), and β_2_: −9.38 (SE: 0.98), resulting in age-specific sensitivities as shown in Figure 3. The sensitivity at 2 years of age was estimated to 0.27, increasing to 0.54 at 3 years of age, 0.68 at 4 years of age, and the maximum sensitivity at 10 years was estimated to 0.79. The resulting proportions of case cows with HI at different ages are also illustrated in Figure 3. At 2 years of age, 33% of the infected cows that would develop HI had developed it, whereas 94% of infected cows eventually developing HI had it at 5 years of age.

Changing the case definition from requiring that the last 2 last samples were positive instead of just one changed the sensitivity estimates as illustrated with dotted lines in Figure 3.

## Discussion

This study takes an unconventional approach to determine the proportion of detectable infected animals at specific ages. We were interested in cows that would eventually develop HI in the expected lifetime, because this group contains cattle primarily associated with production losses and excretion of bacteria in infectious doses [17], [18], [19]. They are also the animals with notable pathological lesions [20]. Presence or absence of HI is thus a key parameter. We then estimated the proportion of the target population, which progressed to HI within the expected lifetime (i.e. became cases) and that could be detected using the milk antibody ELISA at a given age.

Absence of HI might include cows in other stages of the MAP infection, i.e. animals without immune responses or presence of cell-mediated immune responses only. These animals could be of interest if they would be at risk of becoming “diseased” within their expected life-span. To our knowledge, there is limited evidence suggesting that animals without HI become affected by the infection to a degree where they can be considered diseased in terms of production losses, weight loss etc., but they may excrete MAP and thus constitute a potential risk of transmission of MAP [21], [22], [23]. Therefore, we did not consider those animals further.

There are no perfect tests to identify to which infection state an animal belongs. In order to model or predict the infection dynamics, we need to know the proportion of infected animals that will “develop disease in the expected lifetime”. The animals which remain latently infected may be of limited interest if they do not become infectious, do not experience losses and do not become diseased. We used population-based semi-lifelong data to estimate the proportion of infected animals as the proportion of animals demonstrating MAP specific antibodies, which are indicative of anti-inflammatory responses. Subsequently, we estimated the proportion of animals with anti-inflammatory responses at different ages. These proportions are essential for modeling infection dynamics. Current MAP epidemiological models [11], [12], [24], [25] are based on expert opinions regarding assumptions of the distribution between the different infection stages. These models may thus provide biased epidemiological information. The approach taken in this study could be considered controversial, because case-control studies or latent class diagnostic test evaluations are more common. However, these types of evaluations may result in biased or non-interpretable estimates, which may not be concordant with recommended standards [26], [27].

A potential drawback to the current approach was that the true infection status was not known. However, the cow’s final HI status may be more important than their actual infection status, because HI is indicative of loss of control of the infection [3].This is because a cow with persistent latent infection would not be considered as infected and her inclusion would overestimate the sensitivity of the test. This might not be considered a problem by decision makers, since the animal appears to be able to control the infection and may never become infectious. Therefore, she might as well be considered as ‘non-infected’, or on average a ‘non-risk’.

The underlying age-specific estimates of sensitivity were crucial to the estimation of the proportion of cows with HI. Most previous studies have not estimated age-specific sensitivities and consequently cannot compare to the present study. We have previously estimated that the sensitivity increased from 0.06 at 2 years of age to 0.50 at 5 years of age using a case-control study for a different antibody ELISA test [28]. Here, we estimated that the sensitivity of the ID Screen® Paratuberculosis ELISA test was 0.27 at 2 years of age and 0.74 at 5 years of age (Figure 3). However, the antibody ELISA used previously has been shown to be less sensitive and specific than the ID Screen® Paratuberculosis ELISA test used in the present study (Nielsen and Toft, unpublished data). More importantly, changing the case definition reduced the sensitivities by approximately 10%-point, suggesting that the sensitivities were overestimated. However, the age-specific sensitivities could also be underestimated, because we assumed that all cattle were infected in calfhood. This is a common assumption for MAP infections [29], although recent research suggests that cattle exposed to a contaminated environment as adults may have anti-inflammatory reactions to MAP [30].Also, cows with only the last test positive were categorized as cases by definition, but would never have a positive test prior to becoming a case. Consequently, they would essentially pull the sensitivity estimates down. Overall, it seems likely that the estimates are underestimated rather than overestimated, particularly because most infected animals are expected to develop IgG antibodies.

The specificity of the ID Screen® Paratuberculosis ELISA test was similar to those estimated in other studies [18]. Non-specific reactions using this type of samples may be caused by incorrect labeling of milk samples with samples from infected animals being labeled as originating from a non-infected animal and vice-versa, or carry-over milk present in the collection tube between samples. The proportions of cows with HI may appear to be quite high among most age-groups, e.g. 94% of 5-year-old case cows were estimated to have HI (Figure 3). However, the overall proportion of 5-year old cows is relatively low in the Danish dairy population (Table 1) and consequently not contributing greatly to the overall infection dynamics. In a population with a low age distribution, the tip of the iceberg may be somewhat larger than in a population with a higher age distribution, and thereby contribute significantly more to the uncertainty of the infection dynamics. Appropriate inclusion of this information in mathematical models is essential, because infection dynamics is severely affected by the age distribution and consequently the distribution between infection compartments.

### Conclusion

Specificity and age-specific sensitivities of an antibody ELISA were estimated and used for estimation of the proportion of MAP infected cows developing HI in their expected life-time with HI responses at different ages. This proportion was 0.33 for 2-year old animals and 0.94 for 5-year old cows. From this age on-wards, the proportion of infected animals with HI was high. These proportions can be used to study infection dynamics in epidemiological and economical models by inclusion of the transition to HI, and overall the results can be used for better interpretation of test results.
